# A weighted accumulation test for associating rare genetic variation with quantitative phenotypes

**DOI:** 10.1186/1753-6561-5-S9-S6

**Published:** 2011-11-29

**Authors:** Chuanhua Xing, Glen A Satten, Andrew S Allen

**Affiliations:** 1 Department of Biostatistics and Bioinformatics, Duke University, 2424 Erwin Road, Suite 1102 Hock Plaza, Box 2721, Durham, NC 27710, USA; 2 Duke Clinical Research Institute, Duke University, 2400 Pratt Street, Durham, NC 27705-3976, USA; 3 Centers for Disease Control and Prevention, 1600 Clifton Road, Atlanta, GA 30333, USA

## Abstract

Currently there is a great deal of interest in developing methods for testing the role that rare variation plays in disease development. Here we propose a weighted association test that accumulates genetic variation across a signaling pathway. We evaluate our approach by analyzing simulated phenotype data from an exome sequencing study of 697 unrelated individuals from the Genetic Analysis Workshop 17 (GAW17) data set. Although our weighted approach identifies several interesting pathways associated with phenotype Q1, so does an alternative unweighted accumulation approach. Such a result is not unexpected because there is no systematic relationship between the allele frequency of a variant and its effect on phenotype in the GAW17 simulation model.

## Background

Next-generation sequencing technology allows for the characterization of virtually all of an individual’s genetic variation. Genome-wide association studies (GWAS) have successfully detected hundreds of disease-susceptible loci that harbor common variants. However, the common variants identified so far have explained only a small portion of the genetic risk of most of the diseases studied. Some researchers have argued that this is due in part to rare variants having a larger role in disease etiology than previously suspected. Some recent studies support this reasoning [1-11].

Several approaches have been proposed to analyze rare variants for association with disease. The cohort allelic sums test (CAST) is a simple grouping method that compares the number of affected and unaffected individuals who have variants [4,12,13]. Li and Leal [14] introduced the combined multivariate and collapsing (CMC) method. In CMC, markers in a gene or other unit of analysis are collapsed into one or more indicator variables based on some criteria (e.g., the presence of at least one nonsynonymous mutation within a gene). Because many criteria could be used to define several such indicator variables, Li and Leal proposed a multivariate test using Hotelling’s *T*. Morris and Zeggini [15] proposed an accumulation approach that regresses phenotype on a genetic score, defined as the proportion of sites within the gene or pathway that harbor mutations. Price et al. [16] proposed a variable-threshold approach by finding the maximum *z*-score across all possible values for threshold *T*, assuming that the variants having minor allele frequency under this threshold are more likely to be functional. Madsen and Browning [12] proposed a weighted sum statistic. In this approach, single-nucleotide polymorphisms (SNPs), which are rare among the control subjects, are up-weighted with the goal of giving rare, highly penetrant mutations greater influence on the test statistic.

Here, we propose a weighted group-wise association test that accumulates genetic variation across a signaling pathway. We extend the basic idea behind the Madsen and Browning [12] weighting scheme to quantitative traits. Specifically, genetic markers that are rare among individuals in the center of the phenotypic distribution (those with nonextreme phenotypes) are up-weighted to reflect the assumption that rare genetic variation will tend to have a larger effect on a phenotype. Because the weight is a function of phenotype, as in Madsen and Browning’s study [12], permutation is used to assess statistical significance. In the next section, we detail our approach and highlight its application to phenotype Q1 of the Genetic Analysis Workshop 17 (GAW17) data.

## Methods

Suppose that the number of SNPs in a genetic unit (a signaling pathway or a gene) is *P*. Let *Y_i_*, *i* = 1, 2, …, *N*, be the phenotype for individual *i*. We define *I_ij_* for individual *i* to be the number of minor alleles at SNP *j*. Let *X_i_* be a genetic summary score, which we define as:(1)

where *w_j_* is a weight that is applied to the *j*th SNP.

We evaluate two weighting schemes. In the first scheme, *w_j_* is taken to be 1 for all *j*. Thus rare and common SNPs are treated in the same way, and *X_i_* is the simple sum of the number of minor alleles in the gene or pathway. This approach is similar to that of Morris and Zeggini’s method [15]; they defined a genetic score by the proportion of sites within the gene or pathway that harbored mutations. Because this scheme does not differentially weight SNPs, we refer to it as unweighted.

In the second scheme, we calculate the frequency of nonreference mutations among nonextreme individuals at position *j* as:(2)

where *δ*(*Y_i_*) = 1 when *Y_i_* is within one standard deviation of the mean and *δ*(*Y_i_*) = 0 otherwise. Adding a 1 to the numerator and a 2 to the denominator ensures that the frequency *p_j_* is nonzero, so that the weight used in the second scheme,(3)

remains finite [12]. Note that, with this weight, SNPs that are rare among those individuals whose phenotypes lie within the center of the phenotype distribution will be up-weighted and will have a larger role in the genetic summary score *X_i_*. We refer to this scheme simply as weighted.

For both approaches, once we have defined the genetic score *X_i_*, we assume that it is related to *Y_i_* through the linear model:(4)

where *ε* is an unknown error term. A Wald statistic, , is computed, with the variance estimated using a sandwich estimator [17]. Because the weighted approach uses phenotypic information in defining the weight, we use permutation to assess statistical significance. We note that, in this case, the weight is recomputed for each permuted data set. We use 1 million permutations throughout.

We evaluate our approach using the simulated GAW17 data set. These data are described in detail elsewhere [18]. Although all 200 replicates are analyzed, for illustration purposes, we present results concerning replicate 1 in greater detail. Our analyses focus on one phenotype: quantitative trait Q1. Even though we had access to the answers for the underlying simulation model, our approach, including the characterization of signaling pathways, was developed without reference to these answers.

We characterize gene sets using information from two databases. The first, PharmGKB (https://www.pharmgkb.org/) [19], provides information on 1,400 signaling pathways. Unfortunately, the genes in the GAW17 data set are not well represented in PharmGKB, with only 713 out of 3,205 genes sequenced in the GAW17 data being included in 821 of these pathways. To compensate for this low coverage, we also classify genes by biological process from the Gene Ontology (GO) database (http://www.geneontology.org). Although not defining a signaling pathway, the GO biological process domain classifies genes by their involvement in biological processes and therefore presents an interesting unit over which to accumulate genetic variation. This approach allows us to classify 2,304 out of 3,205 genes into 3,009 biological processes. The GO data are contained in two files: a human genetic association file, dated September 15, 2010, revision 1.1433; and a genetic ontology file, dated September 6, 2010, revision 1.160. We note that in both of these classification schemes (PharmGKB and GO) one gene may be mapped to several pathways or biological processes. A pathway or biological process is taken to be significantly associated with the phenotype if its permutation *p*-value does not exceed the Bonferroni corrected significance threshold 0.05(821 + 3009) ~ 1.3055 × 10^–5^. The entire analysis is repeated using both the weighted and unweighted schemes. Only nonsynonymous SNPs are considered throughout.

## Results

The results of these analyses, applied to replicate 1, can be found in Tables 1 and 2. Pathways (processes) with a dagger are significant using the weighted approach, and pathways (processes) with an asterisk are significant using the unweighted approach. Table 1 presents those PharmGKB signaling pathways that were found to be significant by one of the two (weighted or unweighted) approaches. It is immediately apparent from Table 1 that trait Q1 seems to be related to vascular endothelial growth factor (VEGF). This, of course, is comforting, given that the simulation was designed so that genes affecting Q1 came primarily from this pathway.

**Table 1:** Significant PharmGKB signaling pathways

PharmGKB ID	Pathway name	Permutation *p*-value (weighted approach)	Permutation *p*-value (unweighted approach)
PA164713582†*	Actions of nitric oxide in the heart	6.0 × 10^–6^	<1.0 × 10^–6^
PA164713652†*	VEGF hypoxia and angiogenesis	<1.0 × 10^–6^	6.0 × 10^–6^
PA164728105†*	Signaling events mediated by VEGFR1 and VEGFR2	<1.0 × 10^–6^	<1.0 × 10^–6^
PA164728138†*	S1P3 pathway	<1.0 × 10^–6^	<1.0 × 10^–6^
PA164713890†	Neurophilin interactions with VEGF and VEGFR	<1.0 × 10^–6^	1.7 × 10^–5^
PA164714260†	VEGF binds to VEGFR leading to receptor dimerization	<1.0 × 10^–6^	3.3 × 10^–5^
PA164728144†	VEGFR1-specific signals	<1.0 × 10^–6^	1.70 × 10^–4^
PA164728199†	Integrins in angiogenesis	9.0 × 10^–6^	(a)
PA164728205†*	S1P1 pathway	8.0 × 10^–6^	1.1 × 10^–5^
PA164728223†	HIF-1-alpha transcription factor network	<1.0 × 10^–6^	1.92 × 10^–4^
PA164728227†*	Glypican 1 network	1.0 × 10^–6^	<1.0 × 10^–6^
PA2032†*	VEGF pathway	<1.0 × 10^–6^	<1.0 × 10^–6^

† Pathways (processes) that are significant using the weighted approach.

* Pathways (processes) that are significant using the unweighted approach.

**Table 2:** Significant GO processes

GO term	Description	Permutation *p*-value (weighted approach)	Permutation *p*-value (unweighted approach)
GO:0000186†	Activation of MAP kinase activity, especially during sporulation	<1.0 × 10^–6^	4.0 × 10^–5^
GO:0001569†*	Branching involved in blood vessel morphogenesis	<1.0 × 10^–6^	<1.0 × 10^–6^
GO:0001666†*	Response to lowered oxygen tension	<1.0 × 10^–6^	1.0 × 10^–6^
GO:0001938†*	Up-regulation of endothelial cell proliferation	<1.0 × 10^–6^	<1.0 × 10^–6^
GO:0002040†*	Sprouting angiogenesis	<1.0 × 10^–6^	<1.0 × 10^–6^
GO:0006355†*	Regulation of cellular transcription, DNA-dependent	<1.0 × 10^–6^	<1.0 × 10^–6^
GO:0006916*	Anti-apoptosis	7.5 × 10^–5^	1.0 × 10^–6^
GO:0006940†*	Any process that modulates the frequency, rate, or extent of smooth muscle contraction	<1.0 × 10^–6^	<1.0 × 10^–6^
GO:0006952 †	Defense/immunity protein activity	8.0 × 10^–6^	1.9 × 10^–5^
GO:0007169†*	Transmembrane receptor protein tyrosine kinase signaling pathway	<1.0 × 10^–6^	<1.0 × 10^–6^
GO:0008152†	Metabolic process	3.0 × 10^–6^	2.2 × 10^–5^
GO:0010595†*	Up-regulation of endothelial cell migration	<1.0 × 10^–6^	7.0 × 10^–6^
GO:0030097†	Blood cell formation	<1.0 × 10^–6^	(a)
*GO:0030522†*	Intracellular receptor-mediated signaling pathway	<1.0 × 10^–6^	<1.0 × 10^–6^
GO:0030949†*	Up-regulation of vascular endothelial growth factor (VEGF) receptor signaling pathway	<1.0 × 10^–6^	<1.0 × 10^–6^
GO:0043129†*	Surfactant homeostasis	<1.0 × 10^–6^	1.1 × 10^–5^
GO:0045446†	Endothelial cell differentiation	<1.0 × 10^–6^	(a)
GO:0045745*	Positive regulation of G-protein coupled receptor protein signaling pathway	6.425 × 10^–2^	1.2 × 10^–5^
GO:0048286†*	Lung alveolus development	<1.0 × 10^–6^	5.0 × 10^–6^
GO:0048661†*	Up-regulation of smooth muscle cell proliferation	<1.0 × 10^–6^	<1.0 × 10^–6^
GO:0050927†	Up-regulation of positive chemotaxis	<1.0 × 10^–6^	(a)
GO:0051894†	Up-regulation of focal adhesion formation	<1.0 × 10^–6^	(a)
GO:0055074†	Regulation of calcium ion concentration	<1.0 × 10^–6^	(a)

† Pathways (processes) that are significant using the weighted approach.

* Pathways (processes) that are significant using the unweighted approach.

^a^ Once 100 permuted data sets were found to have a larger Wald statistic for a given process than that observed in the original (unpermuted) data set, that process was deemed nonsignificant and further permutations were not performed.

Table 2 presents GO biological processes that were found to be significant by one of the two (weighted or unweighted) approaches. Although VEGF is clearly implicated through one of the significant GO processes, the overall importance of VEGF is far less clear. This does not suggest that the information encoded in the GO database is somehow inferior to that represented by PharmGKB; it suggests only that the organization of PharmGKB makes the involvement of VEGF more transparent in this particular analysis. Results from the analysis of the other replicates are entirely similar. Almost all of the 200 replicates clearly implicate the VEGF pathway as influencing trait Q1. For example, using the weighted approach, PharmGKB pathway PA2032 was found to be significant in all 200 replicates, whereas GO process GO:0030949 was significant in 195 of 200 replicates. The unweighted approach performed even better, with all 200 replicates finding both PA2032 and GO:0030949 significant.

We informally explored which genes were important in these significant pathways and processes by enumerating the number of times a given gene was present in a significant pathway or process. The genes occurring in the biological processes from the GO data set are compared with the ones in the signaling pathways from the PharmGKB data set. Table 3 presents the top 10 genes with the most representation in the list of significant pathways or processes. From this table we can see that, although the GO and PharmGKB approaches may appear different at the pathway or process level, they seem to identify similar structure at the gene level. The genes *VEGFA*, *FLT1*, *KDR*, *HIF1A*, and *ARNT* are consistently represented both in the significant PharmGKB pathways and the significant GO processes. A comparison of the results in Table 3 with those in Table 4 suggests that the unweighted analysis also gives similar results.

**Table 3:** Ten most frequent genes in significant PharmGKB pathways and GO processes using the weighted approach

PharmGKB	Number of nonsynonomous SNPs	GO	Number of nonsynonomous SNPs
**VEGFA**	2	**FLT1**	20
**FLT1**	20	**KDR**	11
SRC	1	**VEGFA**	2
HSP90AA1	9	KIT	5
**KDR**	11	**ARNT**	9
PRKCA	2	PTK2B	4
**HIF1A**	6	**HIF1A**	6
PTK2	5	SHH	4
SHC1	3	ROR2	2
**ARNT**	9	NRP1	1

Bold denotes genes that were found using both databases.

**Table 4:** Ten most frequent genes in significant PharmGKB pathways and GO processes using the unweighted approach

PharmGKB	Number of nonsynonomous SNPs	GO	Number of nonsynonomous SNPs
**VEGFA**	2	**FLT1**	20
**FLT1**	20	**VEGFA**	2
SRC	1	**KDR**	11
HSP90AA1	9	KIT	5
HIF1A	6	**ARNT**	9
PRKCA	2	PTK2B	4
**KDR**	11	PDGFB	3
SHC1	3	SHH	4
PTK2	5	EGFR	4
**ARNT**	9	ROR2	2

Bold denotes genes that were found using both databases.

## Discussion

We presented two tests (weighted and unweighted) that accumulate genetic variation across a signaling pathway or biological process. In the analyses presented here, we found that the unweighted approach worked as well as, or better than, the weighted approach. We believe that this is strictly due to the structure of this particular simulation, in which the effect sizes of causal SNPs show no trend with the frequency of the causal variant. In situations where rarer SNPs are, in fact, more highly penetrant, we would expect a weighted approach to be more powerful.

In the analyses presented here, an accumulation approach seemed to work well. However, we must offer two important caveats. First, when moving from a gene-based to a pathway-based approach, the power of the approach becomes increasingly dependent on the state of existing biological knowledge and its representation in databases such as PharmGKB and GO. Even though we constructed pathways and biological processes without considering the true simulation model, our results are bound to be an overly optimistic representation of the power of a pathway-based approach. After all, the GAW17 simulation was constructed by accessing the same biological knowledge (although perhaps not the same databases) that we used to construct our pathways. Second, we computed a genetic score by simply summing the number of mutations in a gene or pathway and ignoring the directionality of the effect. This approach will be powerful when mutations lead to a shift in the phenotype in only one direction (as in the GAW17 simulation). However, it is likely that some mutations could lead to higher values of a phenotype and that other mutations could lead to lower values. This is possible even within a gene and becomes even more likely when considering a collection of genes, such as in a signaling pathway.

## Competing interests

The authors declare that there are no competing interests.

## Authors’ contributions

CX, GAS, and ASA designed the study. CX implemented the methods and conducted the analyses. CX, GAS, and ASA wrote the manuscript.
